# Randomized, Controlled Study of Opicapone in Japanese Parkinson's Patients with Motor Fluctuations

**DOI:** 10.1002/mds.28322

**Published:** 2020-10-19

**Authors:** Atsushi Takeda, Ryosuke Takahashi, Yoshio Tsuboi, Masahiro Nomoto, Tetsuya Maeda, Akihisa Nishimura, Kazuo Yoshida, Nobutaka Hattori

**Affiliations:** ^1^ National Hospital Organization Sendai‐Nishitaga Hospital Sendai Japan; ^2^ Department of Cognitive & Motor Aging Tohoku University, Graduate School of Medicine Sendai Japan; ^3^ Department of Neurology Kyoto University Graduate School of Medicine Kyoto Japan; ^4^ Department of Neurology Fukuoka University Hospital Fukuoka Japan; ^5^ Department of Neurology and Clinical Pharmacology Ehime University Graduate School of Medicine Toon Japan; ^6^ Department of Neurology Saiseikai Imabari Hospital Imabari Japan; ^7^ Division of Neurology and Gerontology, Department of Internal Medicine, School of Medicine Iwate Medical University Shiwa Japan; ^8^ Department of Clinical Development Ono Pharmaceutical Co., Ltd. Osaka Japan; ^9^ Department of Neurology Juntendo University Graduate School of Medicine Tokyo Japan

**Keywords:** Japanese, opicapone, Parkinson's disease, randomized controlled trial

## Abstract

**Objectives:**

This placebo‐controlled, randomized study evaluated the efficacy and safety of opicapone 25‐mg and 50‐mg tablets in Japanese levodopa‐treated patients with Parkinson's disease and motor fluctuations.

**Methods:**

Japanese adults (n = 437, age 39–83 years) with Parkinson's disease (United Kingdom Parkinson's Disease Society criteria) received opicapone 25‐mg (n = 145), opicapone 50‐mg (n = 145), or placebo (n = 147) tablets over the double‐blind treatment period (14–15 weeks). The primary efficacy assessment was change in OFF‐time; secondary efficacy assessments included OFF/ON‐time responders (≥1 hour change from baseline), total ON‐time, ON‐time with and without troublesome dyskinesia, and Unified Parkinson's Disease Rating Scale.

**Results:**

The least squares mean (standard error) change in OFF‐time from baseline to the last visit was −0.42 (0.21) hour for the placebo group, −1.16 (0.22) hour for the opicapone 25 mg group, and −1.04 (0.21) hour for the opicapone 50 mg group. The percentage of ON‐time responders, changes in total ON‐time/ON‐time without troublesome dyskinesia, and Unified Parkinson's Disease Rating Scale II (at OFF) all showed statistically significant improvements versus placebo for both opicapone tablet doses (*P* < 0.05). Unified Parkinson's Disease Rating Scale III (at ON) was improved versus placebo in patients who received opicapone 50 mg (*P* < 0.05). Adverse events were more common in patients treated with opicapone 25 mg (60.0%) or opicapone 50 mg (54.5%) versus placebo (48.3%). The most commonly reported adverse event was dyskinesia (placebo, 2.7%; opicapone 25 mg, 9.0%; opicapone 50 mg, 12.4%).

**Conclusions:**

In Japanese patients, both opicapone 25 and 50 mg were significantly more effective than placebo with no dose‐dependent difference in efficacy, and both doses were well tolerated. © 2020 The Authors. *Movement Disorders* published by Wiley Periodicals LLC on behalf of International Parkinson and Movement Disorder Society.

End‐of‐dose motor fluctuations (wearing off) represent a key motor complication of levodopa (l‐dopa) therapy among patients with Parkinson's disease (PD), and they increase in frequency with longer duration of disease.^1^ Inhibition of catechol‐O‐methyl transferase (COMT) is an established strategy to treat end‐of‐dose motor fluctuations and reduce OFF‐time in patients with PD treated with l‐dopa and a DOPA decarboxylase inhibitor. Opicapone (BIAL, Portela & CA, S.A.), a novel, once‐daily, third‐generation COMT inhibitor, helps overcome limitations of entacapone and tolcapone, and provides more sustained COMT inhibition than these agents.^1^ Previous placebo‐controlled, randomized clinical trials have demonstrated that opicapone was generally well tolerated and significantly reduced OFF‐time compared with placebo.^2^, ^3^ Reflecting these positive results, opicapone was included as a “clinically useful” treatment option for motor fluctuations by the updated International Parkinson and Movement Disorder Society Evidence‐Based Medicine Review of treatments for PD motor symptoms.^4^

In Europe, BIAL (Coronada, Portugal) developed the widely available hard capsule formulation, whereas in Japan, a smaller tablet formulation was developed by Ono Pharmaceutical (Osaka, Japan). According to a previous randomized, double‐blind, placebo‐controlled study, the pharmacokinetic profiles of opicapone capsules were similar in Japanese and non‐Japanese populations.^5^ Recent studies of the opicapone tablet formulation in healthy Japanese have demonstrated that opicapone improves l‐dopa availability in Japanese patients with PD in a similar fashion to that of opicapone capsules in non‐Japanese patients.^6^, ^7^ However, results of a phase 1 study in healthy Japanese subjects found differences in pharmacokinetics of the hard capsule and tablet formulation developed for use in Japanese clinical trials.^8^ Therefore, it is important to evaluate the efficacy and safety of opicapone tablets in patients with PD with motor fluctuations in Japanese patients. The present study (double‐blind part of COMFORT‐PD [COMt‐inhibitor Findings from Opicapone Repeated Treatment for Parkinson's Disease]) was designed to investigate the efficacy and safety of opicapone 25‐mg and 50‐mg tablets versus placebo in Japanese l‐dopa‐treated patients with PD and motor fluctuations.

## Patients and Methods

### Study Design

This is a multicenter, placebo‐controlled, randomized, double‐blind, parallel‐group part of the COMFORT‐PD study conducted from January 22, 2016, to August 24, 2018. The COMFORT‐PD study was composed of two parts, including a double‐blind and open‐label study. The double‐blind part was designed to assess the superiority of opicapone 25‐mg and 50‐mg tablets to placebo in Japanese patients with PD and end‐of‐dose motor fluctuations treated with l‐dopa plus a DOPA decarboxylase inhibitor. In addition, the study also assessed the safety and tolerability of opicapone.

The study consisted of a 2‐week screening period and subsequent double‐blind period of up to 19 weeks (Supporting Information Fig. S1). The double‐blind period consisted of a 14‐ to 15‐week treatment period, which included an l‐dopa dose adjustment period (2–3 weeks), an l‐dopa dose maintenance period (12 weeks), and a transfer period (maximum 4 weeks) to a 52‐week open‐label extension period (reported separately). Patients assessed as eligible were assigned to identical oral opicapone 25‐mg, opicapone 50‐mg, or placebo tablets at a ratio of 1:1:1 by permuted block (size 6) method at the start of the double‐blind period with study drug distributed to each medical institution by a unit of block and administered at each medical institution in the order of registration generated. Blinding was implemented by the study drug assignment manager.

Institutional review boards at 72 participating sites in Japan approved the protocol. This study was conducted in accordance with the Declaration of Helsinki and in compliance with the study protocol, relevant Japanese standards and ordinances, and the International Conference on Harmonization Good Clinical Practice Guidelines. All patients provided written informed consent before study participation.

The study was registered at the Japic Clinical Trials Information registry (JapicCTI‐153,112). Qualified researchers may request Ono Pharma to disclose individual patient‐level data from clinical studies through the following website: https://www.clinicalstudydatarequest.com/. For more information on Ono Pharma's Policy for the Disclosure of Clinical Study Data, please see the following website: https://www.ono.co.jp/eng/rd/policy.html

### Study Population

Japanese men or women (age 30–83 years) were eligible to participate if they had a clinical diagnosis of PD, according to the United Kingdom Parkinson's Disease Society Brain Bank Clinical Diagnostic Criteria^9^ for ≥3 years, Hoehn & Yahr stages 1–3 at ON stage, a history of clinical improvement with l‐dopa plus a DOPA decarboxylase inhibitor for 1 year or more, received a stable optimized regimen of three to eight daily doses of l‐dopa plus a DOPA decarboxylase inhibitor and other PD medications for ≥4 weeks before screening, and had signs of end‐of‐dose motor fluctuations for ≥4 weeks before the screening period, with a mean total awake OFF‐time (state of akinesia or decreased mobility) of ≥1.5 hours, excluding morning akinesia. Further, at the start of the treatment period (baseline), patients were included in the treatment period if they also had completed self‐rating diary charts in accordance with the instructions and had ≤3 errors per day in the 3 days before treatment baseline, ≥1.5 OFF‐time hours per day while awake (excluding the OFF time before onset of response to the day's first dose of l‐dopa plus a DOPA decarboxylase inhibitor taken on or after wakening), as recorded in the 3‐day symptom diary for 2 of the 3 days before the baseline visit, and acceptable laboratory test results during the screening period.

Key exclusion criteria considered during the screening period included nonidiopathic PD, dyskinesia disability score >3 on item 33 of the Unified Parkinson's Disease Rating Scale (UPDRS),^10^ severe and/or unpredictable OFF periods, treatment with prohibited medications (including entacapone, neuroleptics, venlafaxine, monoamine oxidase inhibitors [except selegiline up to 10 mg daily in oral formulation], or antiemetics with antidopaminergic action [except domperidone] within a month before the start of the screening period), dosage change of concomitant anti‐PD drugs within 4 weeks before the start of the screening period, previous or planned surgery or deep brain stimulation for PD, medical or psychiatric conditions that might interfere with assessments (including dementia, unstable cardiovascular disease, or clinically relevant liver disease), a history of neuroleptic malignant syndrome, neuroleptic malignant syndrome‐like syndromes, or nontraumatic rhabdomyolysis.

### Study Medications

Study medications (opicapone or placebo) were orally administered as identical film‐coated tablets once daily at bedtime at least 1 hour after the last administration of l‐dopa plus a DOPA decarboxylase inhibitor. During the l‐dopa dose adjustment period, the daily dose of l‐dopa could be reduced if considered necessary (eg, safety concerns), after which the daily dose could be increased again to that used at the baseline. During the dose maintenance phase, the dose regimen was to be kept stable.

### Assessments

The primary efficacy assessment was change in OFF‐time from baseline to weeks 14–15 or the last visit for patients who discontinued early. OFF‐time was obtained from a patient symptom diary recorded at 30‐minute intervals during the day for 3 consecutive days before each visit. The diary was completed by patients, with support from family or a caregiver if needed. The mean value of 3 days' OFF‐time before each visit was used, but if data were missing for 1 or 2 of the 3 days, data for the remaining day(s) were used. If OFF‐time data were missing for all 3 days, the symptom diary OFF‐time data were regarded as missing for that visit.

Secondary efficacy assessments were mainly total ON‐time and ON‐time with and without troublesome dyskinesia (all based on patient symptom diary records). ON‐ and OFF‐time responders were defined as patients whose ON‐ or OFF‐time was increased or reduced, respectively, by a predefined duration of 1 hour or more from baseline. In addition, data on UPDRS I (Mentation, Behaviour and Mood), II (Activities of Daily Living), and III (Motor Examination) were obtained. UPDRS I and III were assessed at ON stage, whereas UPDRS II (Activities of Daily Living) was assessed during the ON and OFF stages. Finally, data were gathered in relation to the Modified Hoehn & Yahr Staging at ON stage,^11^ Schwab and England Activities of Daily Living Scale at ON and OFF stages,^12^ Clinician and Patient Global Impression of Change using a scale from 1 = very much improved to 7 = very much worse (score 0 = not assessed),^13^ the 39‐item Parkinson's Disease Questionnaire,^14^ the percentage of patients with decreased l‐dopa daily dose, and the decrease in l‐dopa dose amount.

Safety was assessed regularly via monitoring of adverse events, laboratory tests (hematology, blood biochemistry, urinalysis and blood coagulation), and physical and neurological examinations, as well as determination of body weight and cardiovascular parameters (blood pressure, pulse rate, 12‐lead electrocardiogram). Adverse events were tabulated by System Organ Class and Preferred Term according to MedDRA Version 20.1 (Japanese version). Adverse events were classified according to severity and causal relationship to study medications with adverse events classified as at least possibly related to study medications considered as drug‐related adverse events. Finally, suicide risk was assessed using the Columbia Suicide Severity Rating Scale during the treatment period.^15^

### Statistical Analysis

Populations analyzed included the full analysis set and the safety analysis set. The primary efficacy variable, the change in OFF‐time from baseline to the last visit in the efficacy analysis set, was assessed using an analysis of covariance with treatment group as a factor and baseline OFF‐time as a covariate in the full analysis set. The least squares mean (LSM) of each treatment group and corresponding standard errors (SEs), 95% confidence intervals of LSM of each treatment group, LSM of between‐group difference and corresponding SEs, and 95% confidence interval of LSM for the between‐group difference were calculated. The last observation carried forward method was applied for handling missing data with mixed model for repeated measures and worst observation carried forward methods performed as sensitivity analyses for handling of randomly missing data.

The planned number of subjects was determined to assure the power of the primary analysis (change in OFF‐time from baseline at the last visit) to detect a significant difference assuming a two‐sided significance level (*t* test) of 5%, power of 85%, and standard deviation based on combined results from two overseas phase 3 studies set at 2.56 hours for opicapone 50 mg versus placebo (index case). The hypothesis of this study was that opicapone at doses of 25 and 50 mg is superior to placebo based on the primary analysis. The closed testing procedure was used in the statistical hypothesis testing whereby the opicapone 50 mg group was tested before the opicapone 25 mg group.

For secondary efficacy variables, summary statistics were developed for patient diary and investigator‐assessed items. Frequency distributions of the extent of decrease in symptom diary OFF‐time (<1 hour, ≥1 hour) and extent of increase in symptom diary ON‐time (<1 hour, ≥1 hour) were also developed. The proportion of OFF/ON‐time responders per treatment group was compared using a chi‐square test. Intergroup comparisons were performed on secondary efficacy variables as necessary. Post hoc analyses were also performed in relation to Clinician Global Impression of Change and Patient Global Impression of Change scores at the end of the double‐blind period using Fisher's exact test in addition to the originally planned Wilcoxon's test. The multiplicity of secondary end points was not taken into account from a statistical point of view because these were implemented for exploratory analysis and studying drug efficacy from various perspectives.

Safety analyses were done using the safety analysis set. SAS® software (versions 9.3 and 9.4; SAS Institute Inc., Cary, NC, USA) was used for all statistical analyses.

## Results

### Patient Disposition and Baseline Characteristics

Of 456 patients initially enrolled, 19 patients dropped out during the screening period, and 437 patients were formally registered and randomized (placebo tablet, n = 147; opicapone 25‐mg tablet, n = 145; opicapone 50‐mg tablet, n = 145). Withdrawal from the study was due to patient request or adverse events (Supporting Information Fig. S2).

Baseline characteristics of enrolled patients are summarized in Table 1. Overall, the mean (range) age of patients was 67.4–68.5 (39–83) years, and slightly more than half (58.6%–61.9%) of all patients were female. Patients were generally well matched at baseline with no significant differences between treatment groups.

**TABLE 1:** Baseline characteristics of randomized patients

	Placebo (n = 147)	Opicapone 25‐mg Tablets (n = 145)	Opicapone 50‐mg Tablets (n = 145)
Male sex, n (%)	56 (38.1)	58 (40.0)	60 (41.4)
Age, y, mean (SD)	68.5 (8.6)	67.9 (9.1)	67.4 (7.8)
Body weight, kg, mean (SD)	55.6 (11.2)	56.9 (12.8)	56.9 (13.4)
Duration of PD, y, mean (SD)	7.5 (3.8)	7.6 (3.9)	7.7 (4.9)
OFF‐time, hr, mean (SD)	6.3 (2.6)	5.9 (2.3)	6.0 (2.3)
Total ON‐time, h, mean (SD)	9.9 (2.7)	10.4 (2.7)	10.5 (2.3)
With troublesome dyskinesia	0.1 (0.5)	0.1 (0.4)	0.1 (0.5)
Daily l‐dopa dose, mg, mean (SD)	422.3 (170.1)	407.9 (147.0)	445.3 (175.8)
Daily l‐dopa doses, n, mean (SD)	3.9 (1.2)	3.7 (1.1)	4.0 (1.4)
Concomitant PD medication, n (%)	138 (93.9)	137 (94.5)	140 (96.6)
Dopamine agonist, n (%)	112 (81.2)	121 (88.3)	116 (82.9)
Selegiline, n (%)	65 (47.1)	76 (55.5)	69 (49.3)
Istradefylline, n (%)	34 (24.6)	40 (29.2)	29 (20.7)
Zonisamide, n (%)	38 (27.5)	46 (33.6)	38 (27.1)
Amantadine, n (%)	27 (19.6)	28 (20.4)	30 (21.4)
Anticholinergics, n (%)	15 (10.9)	14 (10.2)	15 (10.7)
Droxidopa, n (%)	8 (5.8)	12 (8.8)	1 (0.7)

Abbreviations: SD, standard deviation; PD, Parkinson's disease; l‐dopa, levodopa.

### Primary Efficacy Variable

**FIG 1:**
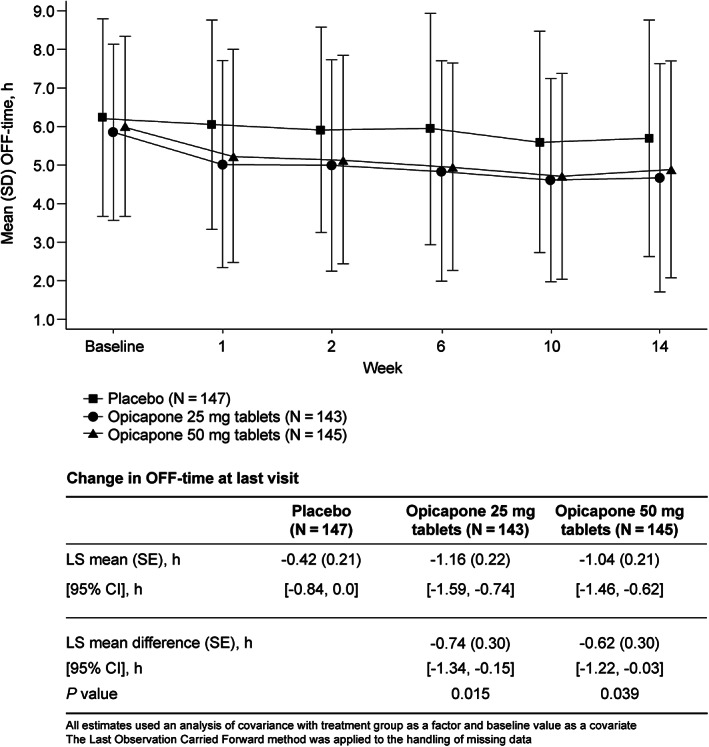
Change over time in mean (standard deviation [SD]) OFF‐time and change in OFF‐time at last visit (bottom table). CI, confidence interval; LS, least squares; SE, standard error.

The LSM (SE) change in OFF‐time from baseline to the last visit was −0.42 (0.21) hour for the placebo group, −1.16 (0.22) hour for the opicapone 25 mg group, and −1.04 (0.21) hour for the opicapone 50 mg group (Fig. 1). The LSM (SE) difference from the placebo group was −0.74 (0.30) hour for the opicapone 25 mg group and −0.62 (0.30) hour for the opicapone 50 mg group (*P* < 0.05 for both opicapone groups). Results of the sensitivity analysis obtained using the mixed model for repeated measures method to handle random missing data, as well as the worst observation carried forward method as an alternate single‐imputation method, led to similar results as those obtained using the last observation carried forward method.

OFF‐time was consistently and steadily reduced in both opicapone tablet groups from week 1 up to the end of the double‐blind part (14–15 weeks) (Fig. 1).

### Secondary Efficacy Variables

Results of secondary efficacy analyses are provided in Table 2. Among the secondary efficacy results, the percentage of ON‐time responders at the last visit, as well as the changes from baseline to the last visit in total ON‐time and ON‐time without troublesome dyskinesia, showed statistically significant differences (*P* < 0.05) compared with placebo for both opicapone doses. Further, the proportion of OFF‐time responders at the last visit also showed a tendency toward improvement with opicapone 25 mg and 50 mg versus placebo (*P* = 0.08 for both comparisons). In addition, the proportion of patients who had their l‐dopa dose reduced was significantly higher for opicapone‐treated patients than placebo‐treated patients (*P* < 0.05 for both doses). The mean value of the l‐dopa dose was almost unchanged throughout the double‐blind period with relative changes in mean l‐dopa dose from baseline level of −0.05%, −0.91%, and −2.04% for placebo, opicapone 25 mg, and opicapone 50 mg, respectively.

**TABLE 2:** Secondary efficacy results presented as change from baseline to last visit or absolute value at last visit

Efficacy Variable	Placebo (n = 147)	Opicapone 25‐mg Tablets (n = 143)		Opicapone 50‐mg Tablets (n = 145)	
	Change or End Value	Change or End Value	*P* Value^a^ vs Placebo	Change or End Value	*P* Value^a^ vs Placebo
Percentage OFF‐time responder ≥ 1 h, n (%)	62 (42.2)	75 (52.4)	0.080	76 (52.4)	0.080
Percentage ON‐time responder ≥ 1 h, n (%)	58 (39.5)	79 (55.2)	0.007	75 (51.7)	0.035
Change in total ON‐time, h, LSM (SE) [95% CI]	0.38 (0.21) [−0.04 to 0.79]	1.14 (0.22) [0.72–1.56]	0.012	1.08 (0.21) [0.66–1.50]	0.020
Change in ON‐time without troublesome dyskinesia,^b^ h, LSM (SE) [95% CI]	0.39 (0.21) [−0.03 to 0.80]	1.11 (0.21) [0.69–1.53]	0.016	1.0 (0.21) [0.58–1.42]	0.041
Change in ON‐time with troublesome dyskinesia, h, LSM (SE) [95% CI]	−0.01 (0.05) [−0.11 to 0.09]	0.03 (0.05) [−0.07 to 0.13]	0.574	0.08 (0.05) [−0.02 to 0.18]	0.237
Change in UPDRS I (at ON), LSM (SE) [95% CI]	−0.1 (0.1) [−0.2 to 0.0]	0.0 (0.1) [−0.1 to 0.1]	0.462	−0.1 (0.1) [−0.2 to 0.1]	0.690
Change in UPDRS II (at OFF), LSM (SE) [95% CI]	−0.6 (0.3) [−1.2 to −0.1]	−1.6 (0.3) [−2.1 to −1.0]	0.018	−1.7 (0.3) [−2.2 to −1.1]	0.010
Change in UPDRS II (at ON), LSM (SE) [95% CI]	−0.1 (0.2) [−0.4 to 0.3]	−0.4 (0.2) [−0.7 to 0.0]	0.232	−0.2 (0.2) [−0.6 to 0.1]	0.484
Change in UPDRS III (at ON), LSM (SE) [95% CI]	−2.4 (0.4) [−3.2 to −1.6]	−3.0 (0.4) [−3.8 to −2.1]	0.330	−3.6 (0.4) [−4.5 to −2.8]	0.040
Parkinson's Disease Questionnaire, LSM (SE) [95% CI]	−2.0 (0.6) [−3.22 to −0.73]	−1.7 (0.6) [−2.97 to −0.46]	0.775	−2.0 (0.7) [−3.29 to −0.69]	0.987
Patients with decreased l‐dopa dose, n (%)	1 (0.7)	7 (4.9)	0.030	13 (9.3)	0.001

The last observation carried forward method was applied to the handling of missing data.

Abbreviations: LSM, least squares mean; SE, standard error; CI, confidence interval; UPDRS, Unified Parkinson's Disease Rating Scale; l‐dopa, levodopa.

^a^ *P* value is for treatment difference (opicapone vs placebo) for change from baseline at last visit or for absolute value at last visit.

^b^ ON‐time without troublesome dyskinesia is ON‐time without dyskinesia and ON‐time with nontroublesome dyskinesia.

Further, compared with placebo, the UPDRS II (at OFF) was significantly improved in patients who received opicapone 25 mg (*P* = 0.0184) and 50 mg (*P* = 0.0098), and the UPDRS III (at ON) was significantly improved in patients who received opicapone 50 mg (*P* = 0.040).

The Clinician Global Impression of Change frequency distributions at the last visit were not significantly different between the placebo and opicapone treatment groups. However, the Clinician Global Impression of Change and Patient Global Impression of Change scores showed a tendency toward improvement in the opicapone groups according to the Wilcoxon test (Table 3). Further, when the Fisher's exact test was applied, the proportion of patients reporting more than minimal improvement (Patient Global Impression of Change) was statistically significant for both opicapone 25 mg (*P* = 0.0248) and 50 mg doses (*P* = 0.0444; Table 3).

**TABLE 3:** Clinician global impression of change and patient global impression of change at last visit

	Clinician Global Impression of Change			Patient Global Impression of Change		
	Placebo (n = 146), n (%)	Opicapone 25‐mg Tablets (n = 143), n (%)	Opicapone 50‐mg Tablets (n = 143), n (%)	Placebo (n = 145), n (%)	Opicapone 25‐mg Tablets (n = 143), n (%)	Opicapone 50‐mg Tablets (n = 143), n (%)
Very much improved	0 (0.0)	5 (3.5)	11 (7.7)	1 (0.7)	2 (1.4)	6 (4.2)
Much improved	28 (19.2)	30 (21.0)	26 (18.2)	16 (11.0)	20 (14.0)	19 (13.3)
Minimally improved	47 (32.2)	48 (33.6)	48 (33.6)	41 (28.3)	54 (37.8)	49 (34.3)
No change	56 (38.4)	49 (34.3)	48 (33.6)	63 (43.4)	45 (31.5)	46 (32.2)
Minimally worse	14 (9.6)	7 (4.9)	7 (4.9)	18 (12.4)	18 (12.6)	16 (11.2)
Much worse	0 (0.0)	4 (2.8)	2 (1.4)	4 (2.8)	2 (1.4)	6 (4.2)
Very much worse	1 (0.7)	0 (0.0)	1 (0.7)	2 (1.4)	1 (0.7)	0 (0.0)
Not assessed	0 (0.0)	0 (0.0)	0 (0.0)	0 (0.0)	1 (0.7)	1 (0.7)
*P* value (Wilcoxon test)	—	0.1589 N.S.	0.0637 N.S.	—	0.0530 N.S.	0.0646 N.S.
More than minimally improved^a^	75 (51.4)	83 (58.0)	85 (59.4)	58 (40.0)	76 (53.5)	74 (52.1)
Less than minimally improved^a^	71 (48.6)	60 (42.0)	58 (40.6)	87 (60.0)	66 (46.5)	68 (47.9)
Not assessed	0 (0.0)	0 (0.0)	0 (0.0)	0 (0.0)	1 (0.7)	1 (0.7)
*P* value (Fisher's exact test)	—	0.2880 N.S.	0.1933 N.S.	—	0.0248^b^	0.0444^b^

The last observation carried forward method was applied to the handling of missing data.

^a^ Calculation of the proportion of patients with “more than minimally improved” or “less than minimally improved” global assessment excluded patients who could not be assessed.

^b^ *P* < 0.05 represents significant change versus placebo.

Abbreviation: N.S., not significant.

The change in the 39‐item Parkinson's Disease Questionnaire scores from baseline to the last visit was also similar between placebo and both opicapone treatment groups (Table 2).

### Safety

Overall, opicapone was generally well tolerated, although adverse events were common and occurred in 48.3%, 60.0%, and 54.5% of patients in the placebo, opicapone 25 mg, and opicapone 50 mg groups, respectively (Table 4). Despite the higher frequency of adverse events in patients receiving active treatment, no adverse event showed a dose‐dependent increase in incidence.

**TABLE 4:** Summary of adverse event frequency

Adverse Event Category, n (%)	Adverse Events			Drug‐Related Adverse Events		
	Placebo (n = 147)	Opicapone Tablets 25 mg (n = 145)	Opicapone Tablets 50 mg (n = 145)	Placebo (n = 147)	Opicapone Tablets 25 mg (n = 145)	Opicapone Tablets 50 mg (n = 145)
Patients with any adverse events	71 (48.3)	87 (60.0)	79 (54.5)	29 (19.7)	49 (33.8)	51 (35.2)
Patients with serious adverse events	2 (1.4)	8 (5.5)	3 (2.1)	0 (0.0)	2 (1.4)	2 (1.4)
Patients discontinued due to adverse events	3 (2.0)	6 (4.1)	9 (6.2)	2 (1.4)	3 (2.1)	7 (4.8)
Adverse events reported in ≥5% of patients in any group						
Dyskinesia	4 (2.7)	13 (9.0)	18 (12.4)	4 (2.7)	13 (9.0)	18 (12.4)
Nasopharyngitis	12 (8.2)	11 (7.6)	5 (3.4)	0 (0.0)	0 (0.0)	0 (0.0)
Hematoma	10 (6.8)	5 (3.4)	6 (4.1)	1 (0.7)	0 (0.0)	0 (0.0)

The most common adverse events (incidence rate ≥ 5% in any treatment group) were dyskinesia, nasopharyngitis, and hematoma, and were mild or moderate in severity in most patients (Table 4). Serious adverse events were also more common in patients treated with opicapone 25 mg (5.5%) and 50 mg (2.1%) compared with placebo (1.4%). All serious adverse events for which a causal relationship was not excluded were resolved or resolving by the end of the double‐blind treatment period.

Other safety‐related measurements, including laboratory tests (hematology, blood biochemistry, urinalysis, and blood coagulation) and cardiovascular and physical assessments, showed no significant change over time or between groups except for a higher change in creatinine phosphokinase among patients treated with opicapone 25‐mg (21.0 U/L) and 50‐mg (20.1 U/L) tablets compared with placebo (4.9 U/L). However, there was no increase in the percentage of patients with abnormal creatinine phosphokinase values among opicapone‐treated patients.

Suicide tendency, as reported by the Columbia Suicide Severity Rating Scale, was higher among patients treated with opicapone 25 mg (3.5%) and 50 mg (2.1%) compared with placebo (0.7%). However, all tendencies consisted of suicidal ideation only, and no suicidal behavior was observed.

## Discussion

In this study, once‐daily adjunct opicapone tablets at doses of 25 and 50 mg significantly reduced OFF‐time compared with placebo among Japanese l‐dopa‐treated patients with PD and motor fluctuations. The secondary efficacy results also supported the primary efficacy results, especially in relation to improvements in ON‐time relative to placebo with most of the increase in ON‐time consisting of ON‐time without troublesome dyskinesia. Further, the proportions of ON‐time responders and, to a lesser extent, OFF‐time responders were greater in opicapone‐treated patients compared with placebo‐treated patients. Importantly, opicapone tablets were well tolerated and associated with few serious adverse events or changes in liver function tests that have been noted with other COMT inhibitors.^16^, ^17^

Overall, the efficacy results in this study are broadly consistent with those seen in previous similar studies conducted in non‐Japanese patients with some notable differences. In the BIPARK‐I/II studies with opicapone capsules, the treatment difference (opicapone vs placebo) was statistically significant only for the opicapone 50 mg dose.^18^ In contrast, although the LSM changes in OFF‐time in this study were numerically lower in Japanese patients than in the BIPARK‐I/II studies for opicapone 25 mg, opicapone 50 mg, and placebo, both opicapone doses were statistically significantly superior to placebo. A previous Japanese dose‐finding trial of the COMT inhibitor, entacapone, similarly found that both opicapone 100 and 200 mg provided equivalent efficacy compared with placebo.^19^ Although the cause of this result was not discussed and is unknown, it does demonstrate that not only opicapone but also another COMT inhibitor in Japan have shown a similar tendency in terms of producing a relatively flat dose response. A previous phase 1 study in Japanese patients showed that the tablet formulation produced higher plasma opicapone exposure than the capsule formulation.^8^ Similar pharmacokinetic profiles and dose dependency were found in Japanese and non‐Japanese patients when administered opicapone capsules in a previous double‐blind study.^5^ However, increase in l‐dopa exposure has been recently shown to reach a plateau after administration of opicapone tablets at doses of 25 mg or higher.^6^ Similarly, the lack of a notable dose‐dependent increase in change in OFF‐time versus placebo in this study may be caused by properties of the tablet formulation rather than characteristics of Japanese patients.

It is also necessary to consider potential differences between Japanese patients enrolled in this study and non‐Japanese patients enrolled in the BIPARK‐I/II studies and their influence on the results when comparing this study with that of the BIPARK‐I/II studies in non‐Japanese patients.^18^ First, as noted elsewhere, pharmacokinetic differences between the tablet formulation used in this study and the capsule formulation used in the BIPARK‐I/II studies may have produced differences in opicapone blood concentration between these study populations. Second, considering that the mean weight of Japanese patients in this study is likely to be considerably lower than in non‐Japanese patients, the plasma exposure to opicapone may have been greater at both the 25 and 50 mg doses in Japanese patients, which may also diminish the dose‐dependent variation in response noted in non‐Japanese patients. Third, there are notable background differences in l‐dopa doses and concomitant PD drugs between these populations. l‐Dopa doses were considerably lower in this study in the BIPARK‐I/II studies both at baseline and at the end of the double‐blind treatment period, which may partly explain the lower numerical changes in OFF‐time observed in Japanese patients. At the time this study was conducted, selegiline was the only selective monoamine oxidase B inhibitor available in Japan, although the rate of concomitant use (approximately 50%) was considerably higher than that of rasagiline in the BIPARK‐I/II studies (11.1%–14.7%). Further, regarding other specific anti‐PD agents, only zonisamide and istradefylline existed in Japan at the time of this study, and so the background treatment is inherently unlike that of the BIPARK‐I/II studies. Finally, the COMT Val158Met polymorphism has been shown to affect the response to entacapone in a double‐blind crossover trial, although no ethnicity‐based comparisons were made.^20^ Although it is possible that such polymorphisms may also affect the dose response to opicapone, this is speculative and has not been confirmed in studies to date. Overall, without specific studies addressing these potential differences, it is difficult to ascertain the reason for the discrepancy between this study and previous studies with regard to dose dependency in efficacy. However, from a practical perspective, the optimal opicapone dose for addressing motor fluctuations in Japan is likely to range from 25 to 50 mg daily.

The main strength of this study was the randomized, double‐blind controlled design, which effectively minimized potential bias between treatment arms. The main limitation of this double‐blind study is that it does not allow assessment of maintained efficacy or evaluation of new safety after long‐term administration.

A long‐term extension phase of this study has been published elsewhere to assess the safety and efficacy of opicapone tablets over periods of up to approximately 1 year.

### Conclusions

Opicapone 25‐mg and 50‐mg tablets were found to be generally well tolerated in Japanese patients with PD and motor fluctuations. Both opicapone tablet doses demonstrated superiority over placebo with no dose dependency between opicapone tablet dosages. From a practical perspective, the optimal opicapone dose for addressing motor fluctuations in Japan is likely to range from 25 to 50 mg daily.

## Author Roles

All authors designed the study, interpreted the data, critically revised and provided final approval for the manuscript, and are accountable for the accuracy of its contents. A.N. and K.Y. conceived the study. A.T., R.T., Y.T., M.N., and N.H. participated in data acquisition. All named authors meet the International Committee of Medical Journal Editors criteria for authorship for this article, take responsibility for the integrity of the work as a whole, and have given their approval for this version to be published.

## Financial Disclosures

A.T. received grants from Ono Pharmaceutical Co., Ltd., Meiji Seika Pharma Co., Ltd., Hisamitsu Pharmaceutical Co. Inc., Pfizer Japan Inc., Sumitomo Dainippon Pharma Co., Ltd., and Kyowa Hakko Kirin Co., Ltd.; and personal fees from Sumitomo Dainippon Pharma Co., Ltd., Kyowa Hakko Kirin Co., Ltd. and AbbVie Inc. R.T. received grants from Ono Pharmaceutical Co., Ltd., Sumitomo Dainippon Pharma Co., Ltd., Eisai Co., Ltd., Sanofi K.K., Pfizer Japan Inc., Novartis Pharma K.K., Takeda Pharmaceutical Co., Ltd., Otsuka Pharmaceutical Co., Ltd., Nihon Medi‐Physics Co., Ltd., Japan Blood Products Organization and Medtronic Japan Co., Ltd.; and personal fees from KAN Research Institute, Inc., Sumitomo Dainippon Pharma Co., Ltd., Takeda Pharmaceutical Co., Ltd., Kyowa Hakko Kirin Co., Ltd., and FP Pharmaceutical Corporation. Y.T. received grants from Ono Pharmaceutical Co., Ltd. M.N. received grants from Ono Pharmaceutical Co., Ltd., and personal fees from Ono Pharmaceutical Co., Ltd., Takeda Pharmaceutical Co., Ltd., Eisai Co., Ltd., Hisamitsu Pharmaceutical Co. Inc., Meiji Seika Pharma Co., Ltd., Sumitomo Dainippon Pharma Co., Ltd., Kyowa Hakko Kirin Co., Ltd., and Kissei Pharmaceutical Co., Ltd. T.M. received other funding from Ono Pharmaceutical Co., Ltd., Sumitomo Dainippon Pharma Co., Ltd., Kyowa Hakko Kirin Co., Ltd., Otsuka Pharmaceutical Co., Ltd., FP Pharmaceutical Corporation, Takeda Pharmaceutical Co., Ltd., Novartis Pharma K.K., AbbVie Inc., and Nippon Boehringer Ingelheim Co., Ltd. N.H. received grants and other funding from Ono Pharmaceutical Co., Ltd.; grants from Japan Agency for Medical Research and Development (AMED), Japan Society for the Promotion of Science (JSPS), and Ministry of Education Culture, Sports, Science and Technology Japan (Grant‐in‐Aid for Scientific Research on Innovative Areas); personal fees from International Parkinson and Movement Disorder Society, Acorda Therapeutics, Inc., Sanofi K.K., Pfizer Japan Inc., Alexion Pharmaceuticals, Inc., Mylan N.V., MSD K.K., and Lundbeck Japan K.K.; personal fees and other from Sumitomo Dainippon Pharma Co., Ltd., Otsuka Pharmaceutical Co., Ltd., Takeda Pharmaceutical Co., Ltd., Kyowa Hakko Kirin Co., Ltd., GlaxoSmithKline K.K., Nippon Boehringer Ingelheim Co., Ltd., FP Pharmaceutical Corporation, Eisai Co., Ltd., Kissei Pharmaceutical Co., Ltd., Nihon Medi‐physics Co., Ltd., Novartis Pharma K.K., Biogen Inc., AbbVie Inc., Astellas Pharma Inc., and Daiichi Sankyo Co., Ltd.; nonfinancial support from IBM Japan, Ltd.; nonfinancial support and other from Boston Scientific Japan K.K. and other from Medtronic, Inc., Mitsubishi Tanabe Pharma Corporation, Hydrogen Health Medical Labo Co., Ltd., ABIST Co., Ltd., Melodian Co., Ltd., Daiwa Co., Ltd., Bayer Yakuhin, Ltd., Nihon Pharmaceutical Co., Ltd., Asahi Kasei Medical Co., Ltd., MiZ Co., Ltd., OHARA Pharmaceutical Co., Ltd., Meiji Seika Pharma Co., Ltd., and Hisamitsu Pharmaceutical Co., Inc. K.Y. and A.N. are employees of Ono Pharmaceutical Co., Ltd.

## Supporting information


**Figure S1.** Study design
**Figure S2.** Patient disposition
