# Supplementary material for: Randomized, Controlled Study of Opicapone in Japanese Parkinson's Patients with Motor Fluctuations
Source: Mov Disord. 2020 Oct 19;36(2):415–23. doi: 10.1002/mds.28322 (PMC7983910; doi:10.1002/mds.28322)

Supplementary Material

Supplementary Figure 1. Study design

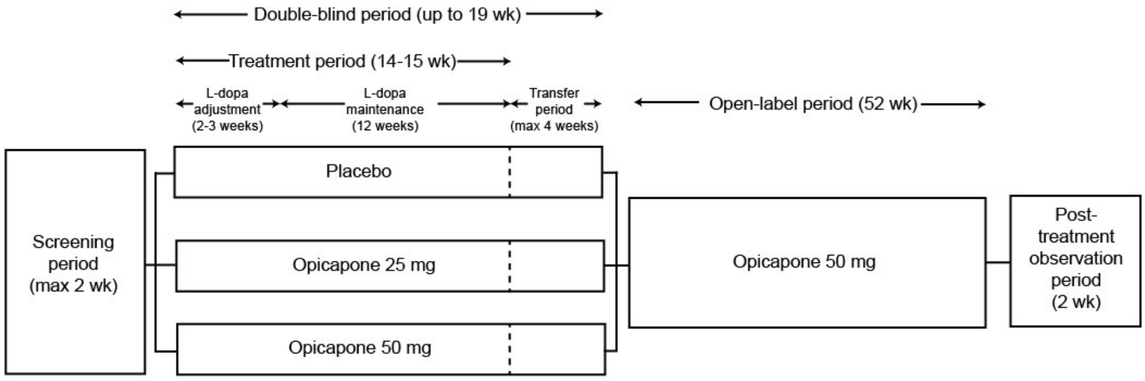

Supplementary Figure 2. Patient disposition

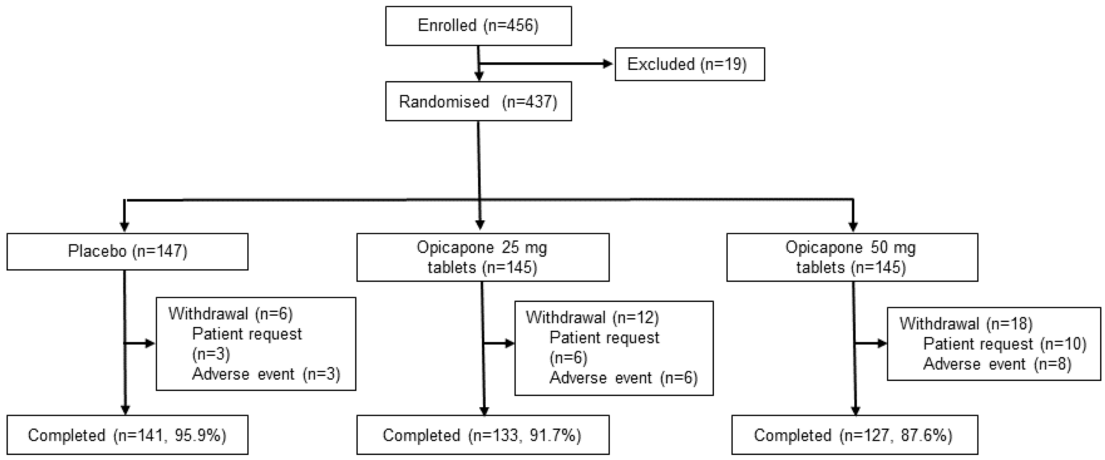

Supplement: Supplementary file 1 — Figure S1. Study design Figure S2. Patient disposition [file MDS-36-415-s001.pdf]
